# Probiotics interventions modulating gut microbiota composition in individuals with intestinal constipation: Protocol of a systemic review and meta-analysis of randomized controlled trials

**DOI:** 10.1371/journal.pone.0311799

**Published:** 2025-01-24

**Authors:** Zubing Mei, Peixin Du, Ye Han, Zhuo Shao, De Zheng

**Affiliations:** 1 Department of Anorectal Surgery, Shuguang Hospital, Shanghai University of Traditional Chinese Medicine, Shanghai, China; 2 Anorectal Disease Institute of Shuguang Hospital, Shanghai, China; 3 Department of General Surgery, The First Affiliated Hospital, Naval Medical University, Shanghai, Shanghai, China; NUR International University, PAKISTAN

## Abstract

**Introduction:**

Intestinal constipation is a substantive global health concern, significantly impairing patient quality of life. An emerging view is that the gut microbiota plays a critical role in intestinal function, and probiotics could offer therapeutic benefits. This study aims to consolidate evidence from randomized controlled trials (RCTs) that assess the effectiveness of probiotics in modulating microbiota and ameliorating symptoms of constipation.

**Methods:**

We will execute a systematic evidence search across Medline (via PubMed), Embase, Cochrane CENTRAL, Web of Science, Scopus, and CINAHL, employing explicit search terms and further reference exploration. Two independent reviewers will ensure study selection and data integrity while assessing methodological quality via the Cochrane Collaboration’s Risk of Bias-2 tool. Our primary goal is to outline changes in microbiota composition, with secondary outcomes addressing symptom relief and stool characteristics. Meta-analyses will adopt a random-effects model to quantify the effects of interventions, supplemented by subgroup analyses and publication bias assessments to fortify the rigor of our findings.

**Discussion:**

This study endeavors to provide a rigorous, synthesized overview of the probiotics interventions evidence for modulating gut microbiota in individuals with intestinal constipation. The insights derived could inform clinical guidelines, nurture the creation of novel constipation management strategies, and direct future research in this field.

**Ethics and dissemination:**

As this study aggregates and analyzes existing data without direct human subject involvement, no ethical approval is required. We will disseminate the study’s findings through scientific forums and seek publication in well-regarded, peer-reviewed journals.

**Trial registration:**

**OSF registration number:** 10.17605/OSF.IO/MEAHT.

## Introduction

Intestinal constipation is a globally prevalent gastrointestinal disorder, affecting an estimated 12–17% of the general population [1]. Its prevalence is considerably higher among older adults, women, and individuals with lower socioeconomic status [2–6]. Intestinal constipation imposes a significant burden on healthcare costs and reduces patients’ quality of life [7–9]. It is characterized by infrequent or difficult passage of stools, often accompanied by bloating, abdominal pain, and a sense of incomplete evacuation. Although the etiology of constipation is multifactorial, recent studies have highlighted the role of gut microbiota in its pathophysiology and potential treatment strategies [10–15].

Gut microbiota comprises a diverse community of microorganisms that play an essential role in the host’s health, including digestive tract functionality, metabolism, and immune system modulation [16]. Dysbiosis of gut microbiota is associated with numerous gastrointestinal disorders, such as inflammatory bowel disease, irritable bowel syndrome, and intestinal constipation [17]. It is postulated that alterations in gut microbiota composition might influence gut motility, secretion, inflammation, and visceral sensation, leading to constipation [18]. Recent findings also indicate that a high-fiber diet can modulate the gut microbiota composition, improving bowel movements and alleviating constipation symptoms [19–22].

Probiotics, defined as live microorganisms that confer health benefits to the host when administered in adequate amounts, have drawn considerable attention for their potential role in alleviating intestinal constipation [23]. The therapeutic mechanism of probiotics in constipation includes their ability to modulate gut microbiota composition, enhance gut motility, increase short-chain fatty acid (SCFA) production, and reduce local inflammation [24]. Probiotics such as *Bifidobacterium*, *Lactobacillus*, and *Saccharomyces boulardii* have been studied in various randomized controlled trials (RCTs) for their efficacy in treating constipation and improving patients’ quality of life [25–27]. Optimal use of probiotics typically involves consistent daily intake over a period of at least 4 to 12 weeks, with evidence suggesting that longer-term use (up to 1 year) may be beneficial for sustained symptom relief. However, while generally safe, probiotics may cause mild side effects such as bloating or gas in some individuals, particularly in the initial phase of treatment. A number of recent meta-analyses have generated mixed results concerning the impact of probiotic interventions on constipation outcomes [25,28,29].

As the body of evidence regarding probiotics’ effectiveness in treating intestinal constipation is growing, an updated and comprehensive systematic review and meta-analysis are warranted to evaluate their overall efficacy and safety systematically. Moreover, analysis of various probiotic strains and dosages, as well as the gut microbiota alterations associated with their administration, might contribute to a better understanding of the subject and help address emerging controversies.

The purpose of this study is to systematically review and meta-analyze RCTs investigating the impact of probiotics interventions on gut microbiota composition and clinical outcomes among individuals with intestinal constipation. The study aims to examine the efficacy and safety of various probiotic strains and treatment regimens, as well as the changes in gut microbiota composition related to these interventions. In addition, our study acknowledges the age-related changes in gut microbiota, such as reduced microbial diversity and variations in specific bacterial populations, which can be pivotal in the onset and persistence of constipation in older adults. If possible, the study will also examine whether certain probiotics can ameliorate these changes, and if so, their efficacy in relieving constipation symptoms. Furthermore, we will introduce the current understanding of the gut-brain axis and its significant influence on gastrointestinal motility and function. We will discuss how dysbiosis may lead to constipation via disruption of this communication axis and the potential modulating role of probiotics.

## Methods

### Study registration

Prior to initiating the literature search, the study protocol was registered in the OSF database (Open Science Framework) under the registration DOI of https://doi.org/10.17605/OSF.IO/MEAHT. OSF registration promotes transparency, accessibility, and adherence to evidence-based medicine best practices by publicly documenting the research question, methods, and any amendments to the study plan, which can be accessed via the website of https://osf.io/meaht. The amendments made will be contemporaneously recorded with full transparency.

The protocol, as well as the subsequent systematic review and meta-analysis, will follow the Preferred Reporting Items for Systematic Reviews and Meta-Analyses Protocols (PRISMA-P) guidelines [30] and Preferred Reporting Items for Systematic Reviews and Meta-Analyses (PRISMA) guidelines [31], respectively, which ensure comprehensive and consistent reporting of all systematic review and meta-analysis components (**S1 Table**). The review team will adhere strictly to the PRISMA guidelines, employing the PRISMA checklist for systematic evaluation at each stage of the meta-analysis.

### Literature searches

To identify relevant randomized controlled trials (RCTs) that investigate the effects of probiotics on gut microbiota composition in individuals with intestinal constipation, we will perform a comprehensive literature search across several electronic databases. The databases we will search include Medline (via PubMed), Embase, Cochrane CENTRAL, Web of Science, Scopus, and CINAHL, ensuring a broad and comprehensive capture of relevant studies [32,33]. We will include studies published up until August 2024, with no restriction on the date of publication.

Our search strategy will combine Medical Subject Headings (MeSH) and free-text terms with Boolean operators to ensure a comprehensive and sensitive search. The detailed search strategies for Medline (via PubMed), Embase, and Cochrane CENTRAL database are presented in **Tables 1–3**. The search terms will primarily be focused on probiotics, gut microbiota composition, intestinal constipation and randomized controlled trial, and the corresponding synonyms and related terms. Our search query will be adapted to the specific syntax and search fields of each database. Our search strategy has been curated to encapsulate a wide range of terms related to probiotics and constipation to ensure comprehensive coverage reflective of the study’s objectives, in keeping with the PRISMA-P guidelines.

**Table 1:** Search strategies for electronic databases.

Pubmed search strategy
Constipation terms:
1 “Constipation”[Mesh]
2 (impaction or delayed bowel movement or obstipation or costiveness or defecation or bowel function* or bowel habit* or bowel movement* or bowel symptom* or bowel motility or colon transit or evacuation or intestinal motility or stool*)[Title/Abstract]
3 1 or 2
Probiotics terms:
4 “Probiotics”[Mesh]
5 “Synbiotics”[Mesh]
6 “Lactobacillus”[Mesh]
7 “Bifidobacterium”[Mesh]
8 “Streptococcus thermophilus”[Mesh]
9 “Lactococcus”[Mesh]
10 “Bacillus subtilis”[Mesh]
11 “Enterococcus”[Mesh]
12 “Enterococcus faecium”[Mesh]
13 “Enterococcus faecalis”[Mesh]
14 “Saccharomyces”[Mesh]
15 (probiotic* or andomize* or lactobacill* or bacill* or bifidus or bifidobacter* or streptococcus thermophilus or streptococc* or lactococc* or bacillus subtilis or saccharomyc* or Leuconostoc or pediococc* or andomize bacillus or beneficial bacter* or Escherichia coli or “E. coli” or Yeast or fungus or fungi or VSL# 3 or VSL3) [Title/Abstract]
16 4-15/or
Gut microbiota terms
17 “Gastrointestinal Microbiome”[Mesh]
18 “Dysbiosis”[Mesh]
19 (microbiota or microbiome or bifido* or lactobacill*) [Title/Abstract]
20 ((faecal or fecal) and (bacteri* or flora)) [Title/Abstract]
21 17-20/or
Study design terms:
22 randomized controlled trial[Publication Type] OR controlled clinical trial[Publication Type]
23 (Randomized or andomized or placebo or drug therapy or randomly or trial or groups) [Title/Abstract]
24 22 or 23
Final search results: Combining Constipation and Probiotics and Gut microbiota and Study design:
15 3 and 16 and 21 and 24

**Table 2:** Search strategies for electronic databases.

Embase search strategy
Constipation terms:
1 ‘constipation’/exp
2 (impaction or delayed bowel movement or obstipation or costiveness or defecation or bowel function* or bowel habit* or bowel movement* or bowel symptom* or bowel motility or colon transit or evacuation or intestinal motility or stool*):ab,ti
3 1 or 2
Probiotics terms:
4 ‘probiotic agent’/exp
5 ‘synbiotic agent’/exp
6 ‘lactobacillus’/exp
7 ‘bifidobacterium’/exp
8 ‘streptococcus thermophilus’/exp
9 ‘lactococcus’/exp
10 ‘bacillus subtilis’/exp
11 ‘enterococcus’/exp
12 ‘enterococcus faecium’/exp
13 ‘enterococcus faecalis’/exp
14 ‘saccharomyces’/exp
15 (probiotic* or synbiotic* or lactobacill* or bacill* or bifidus or bifidobacter* or ‘streptococcus thermophilus’ or streptococc* or lactococc* or ‘bacillus subtilis’ or saccharomyc* or Leuconostoc or pediococc* or ‘bulgarian bacillus’ or ‘beneficial bacter*’ or ‘Escherichia coli’ or ‘E. coli’ or Yeast or fungus or fungi or VSL3):ab,ti
16 4-15/or
Gut microbiota terms
17 ‘intestine flora’/exp
18 ‘dysbiosis’/exp
19 (microbiota or microbiome or bifido* or lactobacill*):ab,ti
20 ((faecal or fecal) and (bacteri* or flora)):ab,ti
21 17-20/or
Study design terms:
22 (‘randomized controlled trial’ OR ‘controlled clinical trial’):it
23 (Randomized or randomised or placebo or ‘drug therapy’ or randomly or trial or groups):ab,ti
24 22 or 23
Final search results: Combining Constipation and Probiotics and Gut microbiota and Study design:
15 3 and 16 and 21 and 24

**Table 3:** Search strategies for electronic databases.

Cochrane Library search strategy
Constipation terms:
1 MeSH descriptor: [Constipation] explode all trees
2 (impaction or delayed bowel movement or obstipation or costiveness or defecation or bowel function* or bowel habit* or bowel movement* or bowel symptom* or bowel motility or colon transit or evacuation or intestinal motility or stool*):ti,ab,kw (Word variations have been searched)
3 1 or 2
Probiotics terms:
4 MeSH descriptor: [Probiotics] explode all trees
5 MeSH descriptor: [Synbiotics] explode all trees
6 MeSH descriptor: [Lactobacillus] explode all trees
7 MeSH descriptor: [Bifidobacterium] explode all trees
8 MeSH descriptor: [Streptococcus thermophilus] explode all trees
9 MeSH descriptor: [Lactococcus] explode all trees
10 MeSH descriptor: [Bacillus subtilis] explode all trees
11 MeSH descriptor: [Enterococcus] explode all trees
12 MeSH descriptor: [Enterococcus faecium] explode all trees
13 MeSH descriptor: [Enterococcus faecalis] explode all trees
14 MeSH descriptor: [Saccharomyces] explode all trees
15 (probiotic* or synbiotic* or lactobacill* or bacill* or bifidus or bifidobacter* or streptococcus thermophilus or streptococc* or lactococc* or bacillus subtilis or saccharomyc* or Leuconostoc or pediococc* or bulgarian bacillus or beneficial bacter* or Escherichia coli or "E. coli" or Yeast or fungus or fungi or VSL3):ti,ab,kw (Word variations have been searched)
16 4-15/or
Gut microbiota terms
17 MeSH descriptor: [Gastrointestinal Microbiome] explode all trees
18 MeSH descriptor: [Dysbiosis] explode all trees
19 (microbiota or microbiome or bifido* or lactobacill*):ti,ab,kw (Word variations have been searched)
20 ((faecal or fecal) and (bacteri* or flora)):ti,ab,kw (Word variations have been searched)
21 17-20/or
Study design terms:
22 (randomized controlled trial OR controlled clinical trial):pt (Word variations have been searched)
23 (Randomized or randomised or placebo or drug therapy or randomly or trial or groups):ti,ab,kw (Word variations have been searched)
24 22 or 23
Final search results: Combining Constipation and Probiotics and Gut microbiota and Study design:
25 3 and 16 and 21 and 24

Additionally, we will manually search the reference lists of all included studies and relevant systematic reviews to identify additional articles that may have been missed during the electronic database search.

Two independent reviewers will screen the search results by title and abstract for potential inclusion in the study. Any disagreements between the reviewers will be resolved through discussion or, if necessary, consultation with a third reviewer. Following the initial screening, the full texts of the shortlisted studies will be assessed for inclusion based on pre-determined eligibility criteria. To mitigate potential search biases or publication bias and ensure the inclusion of grey literature in our review, we will also include trial registries (the International Clinical Trials Registry Platform [ICTRP] and Clinicaltrial.gov), and grey literature (Europe “OpenGrey”) for relevant ongoing and completed trials not yet published in the databases.

### Eligibility criteria and study selection

In our selection process, using the PICOS framework [34], we will incorporate RCTs focusing on patients with intestinal constipation validated by criteria such as Rome III or IV [35]. Our study will encompass a diverse cohort without restrictions based on age, gender, or ethnicity. Interventions evaluated include probiotic formulations of any strain, composition, and delivery method, whether single or multi-strain, encapsulated or not. Comparators will feature placebo, no intervention, or standard care groups. For outcomes, we’ll examine changes in gut microbiota employing techniques like culture, qPCR, or 16S rRNA sequencing [36], and secondary outcomes will evaluate constipation symptomatology including stool frequency and quality, straining, and abdominal pain. For inclusion, RCTs must have a minimum sample size of 50 participants per study arm, as smaller samples may lack the statistical power necessary to detect significant effects of probiotic interventions under optimal conditions. Additionally, the included studies must demonstrate that the research methodology is feasible under medical supervision and nutritional follow-up of the case for a period of no less than one full year, to ensure long-term effects and safety of probiotic interventions. All RCTs selected will be considered regardless of blinding, publication status, or language, aiming for comprehensive inclusivity.

All RCTs will undergo dual independent review against the standardized set of eligibility criteria. These criteria will cater to potential variances in study designs and demographics, and the Standard Operating Procedures (SOPs) will guide reviewers to ensure consistent application. The specific process is as follows:

The study selection process will be conducted in two stages by two independent reviewers following a comprehensive literature search. In the first stage, the reviewers will screen the titles and abstracts of the identified records using the eligibility criteria. Conflicting opinions will be resolved through discussion, and if required, a third reviewer will be consulted. In the second stage, the full-text articles that meet the criteria will be assessed for potential inclusion in the systematic review. Reasons for including and excluding each study at this stage will be explicitly documented and presented using a PRISMA flow diagram [31]. Throughout the study selection process, inter-rater reliability will be calculated using Cohen’s Kappa coefficient to ensure agreement between independent reviewers [37].

### Data extraction

For the eligible studies included in the systematic review and meta-analysis, two independent reviewers will employ a standardized data extraction form that specifically captures detailed information about study characteristics, interventions, and gut microbiota outcomes, including the type of probiotic, dosing, and treatment duration, as well as the metrics used to determine changes in gut microbiota. Specific parameters for microbiota will include diversity indices, compositional changes, and functional metabolic profiles. Cross-verification techniques and author inquiries will ensure completeness and accuracy of the extracted data.

Data extraction will be performed independently by two reviewers using a standardized form that captures study characteristics, interventions, and gut microbiota outcomes. Specific parameters for microbiota will include diversity indices, compositional changes, and functional metabolic profiles. Cross-verification techniques and author inquiries will ensure completeness and accuracy of the extracted data.

Extracted information will encompass study identifiers, including authorship, year, geographic location, and sponsorship. Detailed demographic data, constipation diagnostic standards, and baseline metrics will be recorded for participants. Probiotic intervention specifics—strains used, doses administered, intervention length, and delivery method—alongside comparator descriptions, will be methodically cataloged. Outcome measures will cover both primary metrics of microbiota changes via established laboratory methods and secondary constipation symptom measures. Furthermore, meticulously gathered data on RCT design features—such as blinding techniques and randomization procedures—will also be abstracted.

Disagreements during the data extraction process will be resolved through discussion and consensus, or by consulting a third reviewer, if necessary. Any missing or ambiguous data from the included articles will be handled by contacting the corresponding authors for clarification or additional information. Regular team meetings will serve as checkpoints for adherence to PRISMA guidelines, with an audit trail established to maintain a transparent account of the review process.

### Risk of bias assessment

To appraise potential biases in the included RCTs, two independent reviewers will employ the Cochrane Collaboration’s RoB-2 (RoB-2) tool [38,39]. This assessment includes scrutinizing the randomization process for adequacy and concealment, ensuring interventions align with the protocols, and evaluating the impact of any deviations. It also involves assessing biases from incomplete outcome data, examining outcome measurement methods to detect any measurement biases, and checking for selective reporting of results.

Each domain will be rated as "low risk," "some concerns," or "high risk" of bias [39]. Based on these ratings, an overall RoB for each study will be determined. Discrepancies between reviewers in RoB assessment will be resolved through discussion and, if necessary, by eliciting the opinion of a third reviewer. Any study with a high risk of bias will be subject to exclusion or sensitivity analyses. In addition to employing RoB-2 tool for individual RCTs, our analysis will incorporate strategies to uncover and rectify potential sources of bias including, but not limited to, publication bias, such as selective outcome reporting and language bias.

### Grading level of evidence

The quality of evidence for each outcome will be appraised using the Grading of Recommendations, Assessment, Development, and Evaluation (GRADE) approach [40]. This framework will be employed with thoughtful consideration for the variability and complexity inherent in probiotic and gut microbiota research. Each of GRAD’s domains will be examined with this context in mind, ensuring an accurate representation of the evidence quality in our study. The GRADE framework rates the certainty of evidence across four levels: high, moderate, low, and very low. To evaluate the quality of evidence in our analysis, we will apply five criteria, adjusting our confidence in the findings as necessary. We’ll consider the collective risk of bias in studies, disparities in results, the applicability of findings to the intended population, the precision expressed through confidence intervals, and any indications of publication bias that could downgrade or upgrade the summarized evidence.

### Statistical analysis

All statistical analyses will be performed using Stata12.0 software. Continuous outcomes will be presented as mean differences or standardized mean differences, while dichotomous outcomes will be presented as risk ratios, both with 95% confidence intervals. A random-effects model will be used to pool the effect sizes from the included RCTs, as this approach accommodates potential between-study heterogeneity due to factors such as differing probiotic strains and participants’ characteristics [41]. The results will be presented as forest plots displaying individual study estimates, pooled estimates, and 95% confidence intervals. Heterogeneity between studies will be assessed using the Chi-square test, with a p-value of <0.10 deemed statistically significant. The magnitude of heterogeneity will be determined using the I-square (I^2^) statistic, with I^2^ values of 25%, 50%, and 75% indicating low, moderate, and high heterogeneity, respectively [42]. To explore potential sources of heterogeneity, we will conduct subgroup analyses to explore differences in the effectiveness of probiotics based on socioeconomic status and other demographic factors such as probiotic strains used, study settings, and disease severity, gender, and cultural background, which could influence gut microbiota composition and constipation symptomatology. We will also perform subgroup analyses to understand the probiotics’ effects on different age groups, with a special focus on participants above the age of 50. This will enable us to determine if probiotics interventions differentially influence age-related microbiota shifts in older individuals that are linked to constipation. In addition, we plan to conduct focused subgroup analyses by probiotic type, dosage, and duration of intervention. Subgroup analyses will also be performed based on the GRADE quality assessments to explore the nuances of evidence certainty on the study conclusions. These analyses will evaluate how these factors may modulate the effects of probiotics on gut microbiota outcomes. The subgroup analyses will be reported with forest plots for each grouping, highlighting potential stratification effects. Significant interactions will be noted, and heterogeneity between subgroups will be quantitatively assessed.

Additionally, we will perform meta-regression using study-level covariates to identify potential effect modifiers and quantify their impact on the pooled effect estimates [43]. The stability of results and the impact of individual studies on pooled estimates will be assessed using sensitivity analyses. We will examine the influence of studies with a high risk of bias, different probiotic strains, and varying intervention durations on the effect estimates by sequentially excluding one study at a time. Sensitivity analyses will also be conducted by excluding RCTs rated as low quality by GRADE to test the robustness of the pooled outcomes. Publication bias will be assessed using funnel plots and Egger’s linear regression test when at least 10 studies are included in the meta-analysis [44]. If funnel plot asymmetry or Egger’s test (p < 0.10) indicates potential publication bias, we will apply the trim-and-fill method to identify and adjust for any missing studies [45]. Furthermore, an exploration of grey literature will form part of our assessment to mitigate the impact of unpublished data on our synthesized findings. We will address variations in reporting formats and methodologies for constipation symptoms among RCTs by standardizing metrics where possible and transforming data where necessary.

For statistical significance, we will adhere to the conventional alpha level of 0.05 (*P*<0.05). To determine clinical significance, we will evaluate changes in gut microbiota composition against predetermined benchmarks. Clinically significant differences are defined as a consistent percentage change in key bacterial populations, or the presence/absence of specific strains, correlated with functional improvements in constipation symptoms as measured by validated clinical scales and patient-reported outcome measures.

### Amendments

This protocol for this study will be updated or amended if necessary during the review process. Any protocol modifications will be recorded and included in the manuscript’s supplementary materials with a detailed description of their rationale and recorded on the relevant OSF project page.

## Discussion

### Principal findings

The results from this study will synthesize current evidence on the efficacy of probiotics intervention for modulating gut microbiota composition and alleviating constipation symptoms in individuals with intestinal constipation. The analysis will provide effect estimates and identify specific probiotic strains, dosages, and intervention durations that could lead to significant improvements in constipation symptoms and gut microbiota alterations. Our study will also consider social determinants of health that could potentially influence the gut microbiota and the prevalence of constipation across various demographics. The implications of such factors, along with diet, lifestyle, and healthcare practices, will be discussed in the context of probiotics intervention effectiveness. Our findings will be contextualized within the existing literature regarding the gut-brain axis, elucidating how changes in gut microbiota might affect this axis and influence gastrointestinal motility, which will provide a more comprehensive understanding of constipation etiology and the potential of probiotic interventions to ameliorate these effects.

### Comparison with other studies

Previous systematic reviews and meta-analyses have established the potential benefits of probiotics in alleviating constipation, but they have either focused on specific populations, such as children or older adults [46–48], or on specific probiotic strains [49]. Our review will provide a comprehensive assessment of the probiotics’ intervention on overall gut microbiota composition along with individual strains and will evaluate the most up-to-date RCTs extending the scope of previous reviews. Furthermore, this protocol will integrate PRISMA guidelines with specific adjustments, such as detailed probiotics-related subgroup analyses to determine the relationship between intervention characteristics and therapeutic outcomes.

### Potential mechanisms

The probiotics’ impact on gut microbiota composition and its subsequent effects on constipation can be explained by several mechanisms. Possible mechanisms include promoting gut motility and transit time by modulating myoelectric activity [50], increasing stool frequency and improving stool consistency [5]; regulating gut hormones like motilin, serotonin, and peptide YY, modulating tight junctions to enhance intestinal barrier function, and exerting anti-inflammatory effects on the enteric nervous system [51–55].

### Strengths and limitations

Strengths of this study include a robust and comprehensive search strategy, duplicate review, and assessment processes, and rigorous quality assessment using RoB-2 and GRADE. Additionally, the systematic review will encompass multiple probiotic strains and interventions to provide a comprehensive understanding of their effects on constipation symptoms and gut microbiota alterations.

Potential limitations may arise from inconsistencies in the methodologies of individual studies, such as varying dosages, intervention durations, participant characteristics, and outcome measurements. Other limitations include the possibility of publication bias and the likelihood of unmeasured confounding factors within individual RCTs. However, the presence of publication bias will be assessed and considered in the interpretation of our analysis, with sensitivity analyses helping validate the robustness of our findings against the prior limitations noted in other meta-analyses. Limitations of the GRADE approach in the context of evaluating probiotics research will also be acknowledged, particularly concerning the diversity of study designs and biological variability in gut microbiota outcomes. Our multidisciplinary team expertise has been leveraged to interpret the GRADE assessment critically.

### Conclusions and policy implications

This study will provide evidence-based insights into the effectiveness of probiotics for modulating gut microbiota composition and alleviating constipation symptoms in individuals with intestinal constipation. If proven effective, probiotics could be considered as a complementary and alternative therapy for constipation management. Healthcare professionals, policymakers, and regulatory agencies can use these findings to inform treatment guidelines and clinical practice.

## Supporting information

S1 Table PRISMA-P checklist. (DOCX)

S1 File (DOCX)

S2 File (DOCX)
