# Supplementary material for: Probiotics interventions modulating gut microbiota composition in individuals with intestinal constipation: Protocol of a systemic review and meta-analysis of randomized controlled trials
Source: PLoS One. 2025 Jan 24;20(1):e0311799. doi: 10.1371/journal.pone.0311799 (PMC11759984; doi:10.1371/journal.pone.0311799)
Supplement: S1 File — (DOCX) [file pone.0311799.s002.docx]

**Supplementary Material. PRISMA Flow Diagram**

The following PRISMA flow diagram illustrates the selection process for the systematic review and meta-analysis. The diagram is designed to clearly present each step of the study selection process.

**Identification**

Records identified through database searching (n = XXX)

Additional records identified through other sources (n = XXX)

**Screening**

Records after duplicates removed (n = XXX)

Records screened (n =XXX)

Records excluded (n = XXX)

**Eligibility**

Full-text articles assessed for eligibility (n = XXX)

Full-text articles excluded, with reasons (n = XXX)

**Inclusion**

Studies included in qualitative synthesis (n = XXX)

Studies included in quantitative synthesis (meta-analysis) (n = XXX)

**Figure 1: PRISMA Flow Diagram for Study Selection**
