# Supplementary material for: Probiotics interventions modulating gut microbiota composition in individuals with intestinal constipation: Protocol of a systemic review and meta-analysis of randomized controlled trials
Source: PLoS One. 2025 Jan 24;20(1):e0311799. doi: 10.1371/journal.pone.0311799 (PMC11759984; doi:10.1371/journal.pone.0311799)
Supplement: S2 File — (DOCX) [file pone.0311799.s003.docx]

**Tables and legands**

**Table 1-A. Search strategies for electronic databases**

**A. Pubmed search strategy**

***Constipation terms:***

1 "Constipation"[Mesh]

2 (impaction or delayed bowel movement or obstipation or costiveness or defecation or bowel function* or bowel habit* or bowel movement* or bowel symptom* or bowel motility or colon transit or evacuation or intestinal motility or stool*)[Title/Abstract]

3 1 or 2

***Probiotics terms:***

4 "Probiotics"[Mesh]

5 "Synbiotics"[Mesh]

6 "Lactobacillus"[Mesh]

7 "Bifidobacterium"[Mesh]

8 "Streptococcus thermophilus"[Mesh]

9 "Lactococcus"[Mesh]

10 "Bacillus subtilis"[Mesh]

11 "Enterococcus"[Mesh]

12 "Enterococcus faecium"[Mesh]

13 "Enterococcus faecalis"[Mesh]

14 "Saccharomyces"[Mesh]

15 (probiotic* or synbiotic* or lactobacill* or bacill* or bifidus or bifidobacter* or streptococcus thermophilus or streptococc* or lactococc* or bacillus subtilis or saccharomyc* or Leuconostoc or pediococc* or bulgarian bacillus or beneficial bacter* or Escherichia coli or "E. coli" or Yeast or fungus or fungi or VSL# 3 or VSL3) [Title/Abstract]

16 4-15/or

***Gut microbiota terms***

17 "Gastrointestinal Microbiome"[Mesh]

18 "Dysbiosis"[Mesh]

19 (microbiota or microbiome or bifido* or lactobacill*) [Title/Abstract]

20 ((faecal or fecal) and (bacteri* or flora)) [Title/Abstract]

21 17-20/or

***Study design terms:***

22 randomized controlled trial[Publication Type] OR controlled clinical trial[Publication Type]

23 (Randomized or randomised or placebo or drug therapy or randomly or trial or groups) [Title/Abstract]

24 22 or 23

***Final search results: Combining Constipation and Probiotics and Gut microbiota and Study design:***

15 3 and 16 and 21 and 24

**Table 1-B. Search strategies for electronic databases**

**B. Embase search strategy**

***Constipation terms:***

1 'constipation'/exp

2 (impaction or delayed bowel movement or obstipation or costiveness or defecation or bowel function* or bowel habit* or bowel movement* or bowel symptom* or bowel motility or colon transit or evacuation or intestinal motility or stool*):ab,ti

3 1 or 2

***Probiotics terms:***

4 'probiotic agent'/exp

5 'synbiotic agent'/exp

6 'lactobacillus'/exp

7 'bifidobacterium'/exp

8 'streptococcus thermophilus'/exp

9 'lactococcus'/exp

10 'bacillus subtilis'/exp

11 'enterococcus'/exp

12 'enterococcus faecium'/exp

13 'enterococcus faecalis'/exp

14 'saccharomyces'/exp

15 (probiotic* or synbiotic* or lactobacill* or bacill* or bifidus or bifidobacter* or ‘streptococcus thermophilus’ or streptococc* or lactococc* or ‘bacillus subtilis’ or saccharomyc* or Leuconostoc or pediococc* or ‘bulgarian bacillus’ or ‘beneficial bacter*’ or ‘Escherichia coli’ or ‘E. coli’ or Yeast or fungus or fungi or VSL3) :ab,ti

16 4-15/or

***Gut microbiota terms***

17 'intestine flora'/exp

18 'dysbiosis'/exp

19  (microbiota or microbiome or bifido* or lactobacill*) :ab,ti

20 ((faecal or fecal) and (bacteri* or flora)) :ab,ti

21 17-20/or

***Study design terms:***

22 (‘randomized controlled trial’ OR ‘controlled clinical trial’):it

23 (Randomized or randomised or placebo or ‘drug therapy’ or randomly or trial or groups) :ab,ti

24 22 or 23

***Final search results: Combining Constipation and Probiotics and Gut microbiota and Study design:***

15 3 and 16 and 21 and 24 (2920)

**Table 1-C. Search strategies for electronic databases**

**C. Cochrane Library search strategy**

***Constipation terms:***

1 MeSH descriptor: [Constipation] explode all trees

2 (impaction or delayed bowel movement or obstipation or costiveness or defecation or bowel function* or bowel habit* or bowel movement* or bowel symptom* or bowel motility or colon transit or evacuation or intestinal motility or stool*):ti,ab,kw (Word variations have been searched)

3 1 or 2

***Probiotics terms:***

4 MeSH descriptor: [Probiotics] explode all trees

5 MeSH descriptor: [Synbiotics] explode all trees

6 MeSH descriptor: [Lactobacillus] explode all trees

7 MeSH descriptor: [Bifidobacterium] explode all trees

8 MeSH descriptor: [Streptococcus thermophilus] explode all trees

9 MeSH descriptor: [Lactococcus] explode all trees

10 MeSH descriptor: [Bacillus subtilis] explode all trees

11 MeSH descriptor: [Enterococcus] explode all trees

12 MeSH descriptor: [Enterococcus faecium] explode all trees

13 MeSH descriptor: [Enterococcus faecalis] explode all trees

14 MeSH descriptor: [Saccharomyces] explode all trees

15 (probiotic* or synbiotic* or lactobacill* or bacill* or bifidus or bifidobacter* or streptococcus thermophilus or streptococc* or lactococc* or bacillus subtilis or saccharomyc* or Leuconostoc or pediococc* or bulgarian bacillus or beneficial bacter* or Escherichia coli or "E. coli" or Yeast or fungus or fungi or VSL3):ti,ab,kw (Word variations have been searched)

16 4-15/or

***Gut microbiota terms***

17 MeSH descriptor: [Gastrointestinal Microbiome] explode all trees

18 MeSH descriptor: [Dysbiosis] explode all trees

19 (microbiota or microbiome or bifido* or lactobacill*):ti,ab,kw (Word variations have been searched)

20 ((faecal or fecal) and (bacteri* or flora)):ti,ab,kw (Word variations have been searched)

21 17-20/or

***Study design terms:***

22 (randomized controlled trial OR controlled clinical trial):pt (Word variations have been searched)

23 (Randomized or randomised or placebo or drug therapy or randomly or trial or groups):ti,ab,kw (Word variations have been searched)

24 22 or 23

***Final search results: Combining Constipation and Probiotics and Gut microbiota and Study design:***

25 3 and 16 and 21 and 24 (3075)
